# High spatiotemporal-resolution mapping for a seasonal erosion flooding inundation using time-series Landsat and MODIS images

**DOI:** 10.1038/s41598-024-53552-9

**Published:** 2024-02-20

**Authors:** Jingrong Zhu, Yihua Jin, Weihong Zhu, Dong-Kun Lee

**Affiliations:** 1 College of Agriculture, Yanbian University, Jilin, China; 2 College of Geography and Ocean Sciences, Yanbian University, Jilin, China; 3 Jilin Provincial Key Laboratory of Wetland Ecological Functions and Ecological Security, Hunchun, China; 4 Department of Landscape Architecture and Rural System Engineering, Seoul National University, Seoul, Korea

**Keywords:** Climate sciences, Environmental sciences, Environmental social sciences, Solid Earth sciences

## Abstract

Seasonal erosion flooding events present a significant challenge for effective disaster monitoring and land degradation studies. This research addresses this challenge by harnessing the combined capabilities of time-series Landsat and MODIS images to achieve high spatiotemporal-resolution mapping of flooding during such events. The study underscores the critical importance of precise flood monitoring for disaster mitigation and informed land management. To overcome the limitations posed by the trade-off between spatial and temporal resolution in current satellite sensors, we emplyedand theflexible spatiotemporal data fusion (FSDAF) methods to produce synthetic flood images with enhanced spatiotemporal resolutions for mapping by using MODIS and Landsat data from August 29 to September 3, 2016. A comparison was made between flood maps from several post-disaster forecasts based on ground-obtained time-series images of the Tumen River flood in China. According to the FSDAF approach, the input Landsat image of March 25, 2016, and the fused results had a root mean square error (RMSE) of 0.0301, average difference of 0.001, r of 0.941, and structure similarity indexof 0.939, indicating that temporal variation data had been effectively incorporated into a forecast on August 16, 2016. Results also indicated that the FSDAF forecast values are lower than those from the actual Landsat image. The results of the study also showed that the generated images could be effectively used for flood mapping. By using our newly developed simulation model, we were able to produce a comprehensive map of the inundated areas during the event from August 29 to September 3, 2016. This shows that FSDAF holds great potential for flood prediction and study and has the potential to benefit further disaster-related land degradation by combining multi-source images to provide high temporal and spatial resolution remote sensing information.

## Introduction

The looming threat of floods in both rural and urban areas worldwide has become increasingly severe, posing significant challenges to communities and economies. Recent studies have shown that climate change is causing more intense rainstorms than previously predicted^1,2^. This is particularly evident in areas with heavy precipitation, such as the Tumen River Basin. On August 20–31 2016, the region received a record 648 mm (25.5 inches) of rain, resulting in inland and river flooding as well as backwater flooding. According to the United Nations Office on Humanitarian Affairs (OCHA), the heavy rains and floods in North Hamkyung province in North Korea claimed 133 lives and left 395 people missing. Over 35,500 families were affected, with 69% of their homes completely destroyed, and 8000 public infrastructures damaged. Accurate data on the extent of flood damage is crucial to evaluate the effectiveness of flood prevention and management strategies^3^. Mapping crops^4,5^, water^6,7^ and flood with multi-source satellite data enhances agricultural precision and monitoring capabilities. The temporal variation and spatial extent of the affected regions must be understood to effectively mitigate the flood hazard^8,9^. Landsat data was used to map annual spring floods in the St-John River region, New Brunswick, Canada, from 1985 to 2016. The approach involves decision trees and image thresholding, producing seasonal time series of spring and summer water extents. The study suggests improvements, including multi-sensor integration with radar^10^. Sentinel-1 satellite radar imagery was employed to promptly detect riverbank erosion along the Jamuna River in Bangladesh. The approach involves land cover classification and identification of changes from vegetation to sand or water after the monsoon. Settlements on eroded land are identified as persistent scatters. The study highlights the capability of determining erosion locations just 1 month after the monsoon, providing an advantage over optical satellite images. Freihardt and Othmar^11^. The Sentinel-1 SAR images and the bi-temporal image transformer (BiT) method were used to accurately map inundation extents in Poyang Lake during the 2020 flood. BiT, utilizing ResNet and transformer mechanisms, achieves a high F1-score of 95.5% when compared to other CNN-based methods. The analysis highlights peak inundation on July 14th and significant decline by November, with approximately 600 km^2^ of cultivated land affected. Flood hazards in the Ngan Sau and Ngan Pho river basins in North-Central Vietnam were evaluated using GIS and AHP. Analyzing various factors, it identifies distance from rivers and topographic wetness index as the most influential. The flash flood hazard map, validated with 151 sites, shows 84.8% conformity with moderate to very high hazard areas, mainly along main rivers and streams. The study underscores the effectiveness of GIS, AHP, and Landsat-8 remote sensing for flash flood hazard mapping in the region^12^

Remotely sensing information can provide a spatial consistent and continuous representation of the earth’s surface, making it a valuable tool for monitoring soil erosion, and seasonal water surface dynamics. This is particularly relevant for areas where streams and rivers cross international borders, as remote sensing can provide independently verifiable and consistent data^13–17^. Significant efforts had been made to map flood events using coarse-spatial-resolution satellite observations of water dynamics in various regions worldwide^18–20^. NASA has recently released the NASA Near Real Time Global Flood Mapping System, which automatically generates products for almost the entire world on a near real-time basis using MODIS data for reconstructing and deep estimation of High-Spatial-Resolution images^21–23^. However, a significant proportion of water surface changes occur at resolutions lower than the 250/500 m MODIS resolution, making remote sensing information with coarse spatial resolution insufficient to capture detailed water surface variations.

The use of supervised deep learning and time series remote sensing data^24–27^ with Landsat-like resolutions provides a valuable alternative for mapping soil water erosion and water surface dynamics, due to its 30-m spatial resolution on a global scale, long data record since 1972, reliable and consistently calibrated data, and continuing mission with future data availability^28^. Over many years, Landsat images have been used to map variations and seasonal changes at the continent and sub-continent scales. Despite these advantages, obtaining fine image super-resolution (cloud cover < 10%) for a specified period (e.g., a year or a season), can be challenging in many areas due to cloud contamination hazard effect of underground^29^, the Landsat-7 scan line corrector error issue, and the incomplete spatial coverage of global receiving stations before the Landsat-8 launch^28^. This can fail to capture important short-term hydrologic events such as floods. Moreover, the backwaters of new dams can obscure seasonal flooding areas, while downstream fluvial mechanisms become increasingly dry and fragmented, effects that cannot be inferred from infrequent Landsat data. A study introduces an improved method for estimating the cover management factor (C-factor) in erosion modeling, using detailed monthly land use/land cover maps in Central Greece. Leveraging a biophysical index from Sentinel-2 imagery at a 10-m resolution enhances precision. Monthly C-factor computations reveal basin-scale fluctuations, emphasizing the importance of vegetation density seasonality. The findings are reproducible and applicable to all European Union Member States with similar datasets, providing a harmonized continental approach to erosion modelling^30^.

In this study, we implemented a sophisticated time-series approach that capitalizes on coarse-resolution data to achieve precise mapping of rare hydrological events at a heightened resolution. Our innovative method seamlessly integrates Landsat data with time-series information from MODIS, thereby overcoming the traditional limitations associated with the trade-off between spatial and temporal resolutions. By synergizing the strengths of Landsat’s high spatial resolution and MODIS’s frequent temporal coverage, our approach enables the mapping of water surfaces on significantly shorter time scales compared to conventional methods. To rigorously test the effectiveness of our proposed approach, we chose the downstream region of the Tumen River basin as our research area. This particular choice presented unique challenges related to data collection, primarily due to the region's geopolitical complexity, as it borders three countries. Additionally, the lack of readily available comprehensive measurement data from multiple national agencies heightened the intricacy of the study. Overcoming these challenges underscores the robustness and applicability of our approach in scenarios where data accessibility is constrained, emphasizing its potential for addressing critical gaps in hydrological monitoring in transboundary regions.

## Materials and methods

### Study area

**Figure 1:**
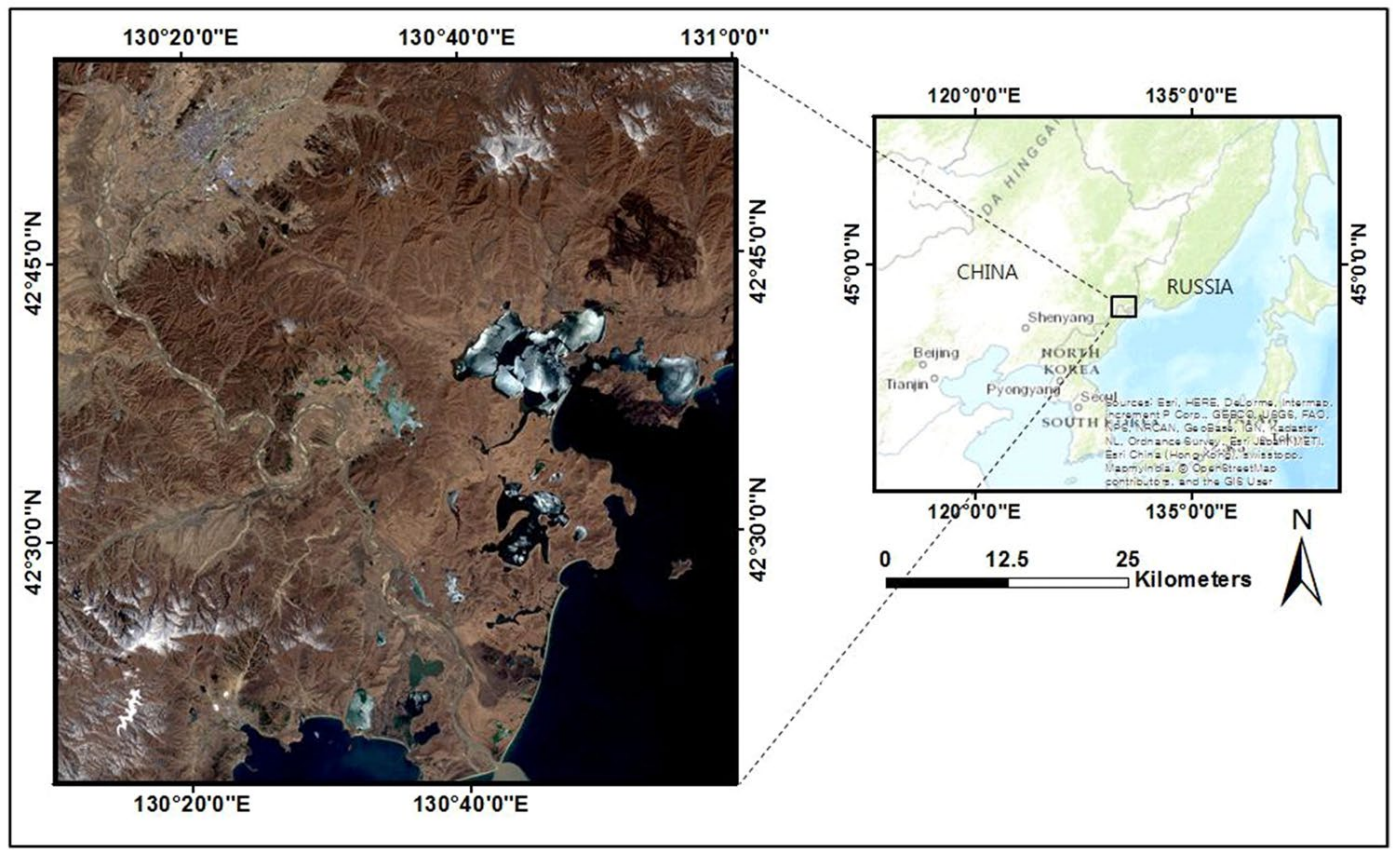
The study area in the Tumen River Basin and the sampling locations are highlighted on the Landsat 8 OLI image captured on March 25, 2016 (displayed in RGB format). The figure was extracted from the Landsat 8 OLI image provided by Geological Survey (https://www.usgs.gov/).

The study area (Fig. 1) is in the mid-western Tumen River Basin in China (42° 30′ N–130° 40′ E). This river basin borders China, Russia, and North Korea along the western coast of the Japanese Sea. The 900 × 900 km^2^ study area encompasses the majority of the city of Hunchun and contains a variety of landscapes, including urban land, water bodies, and vegetation. As previously mentioned, this region experienced a severe flood event in August 2016. It has a typical marine climate with an average annual temperature of 5–6 °C. Along the Tumen River watershed, there are countless man-made and natural oases, many of which have been converted into agricultural land.

### Satellite images and data processing

In this work, the FSDAF was used to create 30-m Landsat-like pictures using a variety of Landsat and MODIS data as inputs. Flexible spatiotemporal data fusion (FSDAF) is an advanced method for improving the spatiotemporal resolution of satellite imagery, particularly beneficial in addressing the inherent trade-off between spatial and temporal resolutions. By integrating data from multiple sources, such as high-spatial-resolution Landsat images and high-temporal-resolution MODIS images, FSDAF captures temporal variations through adaptive weighting techniques and a sophisticated fusion algorithm. This allows the method to intelligently prioritize information based on its relevance to specific temporal and spatial contexts. To provide the necessary results, the FSDAF needs several pairings of Landsat and MODIS photos. To evaluate the use of FSDAF in this study, two cloud-free Landsat pictures and associated MODIS surface reflectance data were acquired. Tow Landsat images taken on July 6 and August 16 were downloaded from USGS database websites (https://earthexplorer.usgs.gov/). Each image’s reflectance was altered to make it similar to MODIS data. The data are of good general quality. The NASA Land Processes Distributed Active Archive Center provided daily 500 m resolution MODIS nadir BRDF (bidirectional reflectance distribution function) calibrated reflectance data groups (MCD43A4) for the same time period. Due to the Tumen River’s constant cloud pollution, the research employed 16-day composite MODIS data rather than common MODIS data. The 16-day composite MODIS data offered a clearer view of the research region and was shown to produce higher-quality synthetic images through mixing approaches^31^. MCD43A4 is a 16-day rolling composite and presents the optimal BRDF reflectance data feasible. Unlike in previous data collections, the information related to the daily data group is centered on the moving 16-day window of the input, rather than its first day.

In total, the maximum inundation border and the Tumen River flood inundation period were tested using six MODIS data sets that were gathered during the flooding event from August 29 to September 3, 2016. The FSDAF model was built using the two Landsat data pictures taken on July 6, 2015, while the image received on August 16, 2016 served as a reference for calculating the model's accuracy. The research used 2016 Landsat-8 OLI photos of the study area with no clouds (less than 10% cloud cover). This research used the Landsat Ecosystem Disturbance Adaptive Processing System (LEDAPS) to calibrate and correct each picture for air disturbance since MODIS utilizes the same radiative transfer technique.

### Forecasting flooding images by FSDAF and accuracy evaluation

By integrating two Landsat pictures, several MODIS images captured on the same day (Tb), and upcoming data, FSDAF created a synthetic image (Tn). There were six major phases in the procedure: dividing the residuals based on the Thin Plate Spline prediction, categorizing the Landsat image at Tb, assessing the temporal variation of each class in the MODIS image from Tb to Tn, forecasting the Landsat image at Tn using the class-level temporal variation, computing the residuals at each MODIS pixel, predicting the Landsat image from the MODIS image at Tn using a Thin Plate Spline interpolator, and obtaining the final Lands Surface data from Landsat and MODIS are very consistent^32^, although there are variances because of variations in solar geometry and bandwidth. A bias element (), which reflects the overall disagreement between the sensors brought on by variations in solar geometry and bandwidth, is added to the total of all Landsat pixels included in a MODIS pixel to determine its value.

### Flood mapping and accuracy assessment

The remote sensing categorization of the region based on single-data images is highly uncertain due to the complex nature of flood inundation and the continuous blending of classes Dronova et al.^33^. The intricate surface composition and dynamics resulted in numerous potential single-variable paths, some of which may not be practically feasible^34^, leading to noise and error^35^.

**Figure 2:**
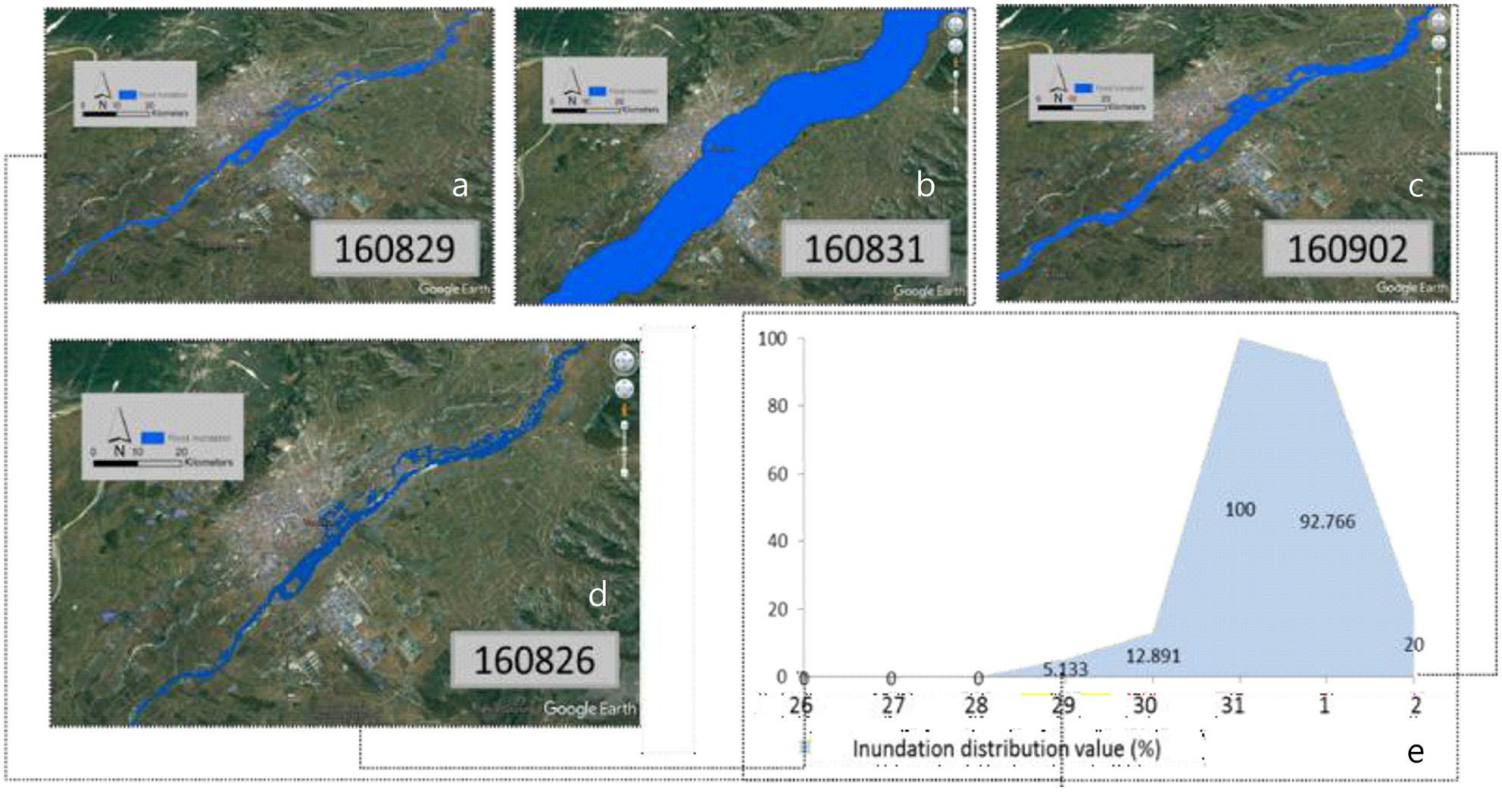
Simulated flood inundation map from August 29 to 3 September 3, 2016. The figure was extracted from the Landsat 8 OLI image provided by Geological Survey (https://www.usgs.gov/).

With minor adjustments, the method given by Sakamoto et al.^18^ served as the foundation for the one utilized in this study. A review of the original method was done, and important components were left out for this study. This research excluded the wavelet-based filter, which was included in the original technique to smooth data by eliminating noise and interpolating missing data. Instead, a decision tree was used in the study to group each pixel into one of four categories: water, flood, mixed, or not connected to floods. Figure 1 depicts the decision tree, which may be summed up as follows: Finding cloud cover pixels in a picture is the first stage. If the blue reflectance (band 3 of MODIS) was below 40.2, pixels were considered to be foggy^36,37^. The photos were then cleaned of the information related to these hazy pixels. The MNDWI, DVEL, LSWI, and the differential value of MNDWI for each land class group were then assessed. To distinguish between pixels that were connected to water and those that weren’t, our study used the technique outlined by Xiao et al.^36^. MNDWI, DVEL, and LSWI were used to differentiate between flood, mixed-flood, and non-flood pixels. The variance in MNDVI, DVEL, and LSI for various land types in 2007 is shown in Fig. 1. A pixel was categorized as not being connected to a flood if it had an MNDWI of 40.3. The MNDWI of permanent water bodies such as “Sea” and “River” land-use categories was > 0.05, and the DVEL of these land categories was > 0.05, indicating that water-related pixels need to have a DVEL of > 0.05. The DVEL value wasn't always > 0.05 for the land-use category “Lake,” however. To address this problem, a different criterion was employed to determine which pixels were associated to water. If the LSWI was 0 and the MNDWI was − 0.05, the pixel was deemed to be related to water. It was crucial to identify whether water-related pixels were flooding pixels, mixed pixels, or long-term water body pixels after water-related pixels had been located. The Landsat satellite sensor’s modest resolution (30 m) made it impossible to discern between vegetation that was partially submerged and vegetation that was completely submerged. Figure 2 demonstrates that MNDWI values for the land categories “River,” “Sea,” or “Lake” were 0.1, indicating that this standard may be used to classify pixels connected to water. If a water-related pixel had an MNDWI > 0.1, it was considered a flooding pixel. If the MNDWI was 40.1 but > 0.3, it was considered to be a mixed pixel. Finally, regions that were submerged for the entire year needed to be separated from mixed and flooding pixels. For example, many water bodies in the Tumen River, such as large wetlands known as “Haors” and small wetlands known as “Beels” were considered submerged.

In order to maximize the distance between the closest points in each class, a Decision Tree (DT) was employed to determine the optimal hyperplane for dividing the flooding and non-flooding classes^38^. DT has been shown to be competitive with other well-known learning algorithms^39^ and is often used in categorization. As training examples, pixels that could be reliably categorized as submerged or not by this approach were chosen. A submersion map was created from each forecast picture using these training samples, combined with the actual image taken at the projected time that served as the flood reference map. Despite not being a genuine depiction, the flood reference map was nonetheless classified using the same training samples as the actual Landsat data. The classifier might be used as a benchmark to assess how well various mixing strategies performed in the flooded map.

### Tumen river flood inundation simulation

The flood event occurred from August 29 to 31, 2016. Thus, the study used August 29, 2016, to September 3, 2016, as the time frame to simulate the flood event and its inundation region. Firstly, the study predicted flooding images through FSDAF during this time frame. Secondly, DT mapping was used to identify both flood inundation and non-inundation areas. To verify the accuracy of the simulation results, the study also utilized flood statistics obtained from the United Nations OCHA. Finally, the study captured the time frame of the flood event, the date, and the region of maximum inundation.

During the flood, the resulting map depicted the flooded areas, which were generally consistent with the actual scenario. Additionally, the maximum inundation area and the most severely flooded spots were analyzed. The dates when the flood started and ended were also recorded.

## Results and discussion

### Test with satellite images in a heterogeneous landscape

The capacity of the FSDAF technique to anticipate Landsat-like pictures has been tested using satellite photographs in different settings, as shown on March 25, 2016. A zoomed-in area was chosen to highlight the differences between the prediction and the actual photographs. The visual comparison reveals that the FSDAF pictures are comparable to the original Landsat image, demonstrating that the method is capable of capturing the temporal changes in croplands from March 25, 2016, to August 16, 2016. As can be seen in the zoomed-in photos, the FSDAF-predicted image is more precise than the images predicted by spatial details. In a single paddy field, a comparison of two source Landsat photos in the zoomed-in area demonstrates a transition from non-vegetation to vegetation. Indicating that the FSDAF technique successfully included temporal variation data to provide a prediction on August 16, 2016, the quantitative index computed from the input Landsat picture of March 25, 2016, and the fused results showed an RMSE of 0.0301, AD of 0.001, r of 0.941, and SSIM of 0.939. A visual comparison between the actual image and the predicted results using the FSDAF method for March 25, 2016, shows that the built-up details remain unchanged with no variation in land overlay categories. In contrast, the paddy and forest forecast by FSDAF are similar in shape to the actual image, demonstrating the ability of FSDAF to generate a realistic imitation of seasonal changes in objects that have undergone land overlay category variance.

### Time series flood inundation

The innovative DT categorization method employed Landsat OLI and combined spectral bands with time sequences from MODIS to simulate the flooding of the Tumen River basin on August 29, 2016. The utilization of two pairs of MODIS and Landsat images facilitated a comprehensive assessment of flood mapping accuracy. Flood maps generated from Landsat images, along with the corresponding predicted (forecast) images using the decision tree method, were analyzed. In these maps, the flood class is visually represented by the color blue, while the non-flooding class is depicted in gray. Discrepancies between the reference map and the flooding map were observed, suggesting potential overestimation of the flood extent.

The temporal analysis of flood inundation revealed intriguing patterns. The red color, indicative of underestimation, was predominantly observed along the peripheries of flooded areas. In contrast, the blue color, representing overestimation, was concentrated near the shores of lakes and riverbanks. Post-flood event forecast maps exhibited larger gray regions compared to the reference map, indicating a substantial disparity between forecasted and observed conditions. This aligns with the earlier analysis of superficial reflectance values.

The assessment of the fusion matrix provided valuable insights into the performance of the mixing techniques employed for generating flooding maps. Table 3 presents key metrics such as overall precision, kappa coefficient, and producer and user accuracy for the flood zone across seven prediction flood maps. The “ua” metric signifies the percentage of pixels on the prediction map correctly identified as flood pixels, while “1-ua” denotes the commission error, revealing instances of overestimation in the forecast maps. On the other hand, “pa” represents the percentage of flood pixels correctly classified in the reference maps, and “1-pa” indicates the omission error, highlighting instances where the flooding zone was underestimated in the prediction maps.

By calculating the averaged difference (Diff) and root mean square error, Table 1 compares the predicted values from FSDAF with the actual Landsat data in the green, red, and NIR bands (RMSE). The important findings of the forecast results are highlighted in the table. First off, there are modest but negative Diffs between the whole Landsat picture and the anticipated image on August 29, 2016, showing that the prediction values produced by FSDAF are lower than those from the actual Landsat image. The fused findings of FSDAF, however, exhibit decreased RMSE values and greater correlation coefficients (r) and structural similarity (SSIM) among the six bands (as seen in Table 1). This conclusion is further supported by scatter plots of actual versus predicted values for the NIR band utilizing FSDAF methods, where the predicted values are almost identical to the actual values. As two Landsat photos were captured throughout the growth season of crops, the NIR band had more reflectance variation than the other bands. FSDAF techniques were able to provide almost unbiased outcomes for each band in terms of overall forecast bias (|AD|b0.000).

**Table 1:** Accuracy assessment of FSDAF fusion approaches employed in the research area with varying land cover categories.

	RMSE	R	AD	SSIM
Band 1	0.014	0.812	0.000	0.891
Band 2	1.022	0.833	0.000	0.900
Band 3	0.034	0.888	0.000	0.802
Band 4	0.066	0.781	0.000	0.731
Band 5	0.044	0.900	0.000	0.911
Band 7	0.035	0.876	0.000	0.893

The units displayed below are reflectance values (*SSIM* structure similarity, *AD* average difference from true reflectance, *RMSE* root mean square error, *r* correlation coefficient).

### Predicted surface reflectance on flood date

The levels of agreement between the categorized predicted pictures and the categorized source Landsat image with the same resolution are shown in Table 2. A stronger connection between the classification map of a chosen prediction picture and the source image is indicated by higher values of kappa and oa. The greatest kappa and oa values are seen in the classification of the FSDAF prediction picture. Additionally, for all four classes, FSDAF has a higher agreement. The FSDAF forecast picture has enhanced pixels that have experienced the land cover category fluctuation during the flood event, namely the drowned area and water. Varied methods are now being developed to combine optical and SAR pictures in order to benefit from SAR images in bad weather and enhance their interpretation in various locations^40,41^. For monitoring floods, several researchers have also employed hybrid fusion techniques^42,43^. Our further research will thus concentrate on integrating SAR pictures into the image fusion framework for the study of urban floods.

**Table 2:** Agreement indexes between predicted image categorizations and categorization of the source Landsat image using the same information.

	2016.08.16	2016.08.29	2016.08.30	2016.08.31	2016.09.01	2016.09.02	2016.09.03
User’s accuracy	0.884	0.840	0.832	0.858	0.932	0.922	0.917
Producers’ accuracy	0.887	0.871	0.889	0.862	0.912	0.901	0.900
Overall accuracy	0.878	0.838	0.864	0.854	0.901	0.921	0.898
Kappa	0.803	0.791	0.805	0.808	0.840	0.833	0.821

Figure 2 shows forecast reflectance images generated by FSDAF. The first image (Fig. 2a) is a Landsat image, followed by three real-time predictions (Fig. 2b–e). A forecast information image (Fig. 2c) is provided as a reference at the bottom. One of the input MODIS images from August 2016 shows a small patch of inundation. However, compared to other forecasts, Fig. 2d is the most affected. To demonstrate the different performances of all trials in flooding regions, two sub-areas, a community in Hunchun and a freeway intersection, are highlighted (as indicated in Fig. 2a) The total real-time forecasts show a sudden drop in reflectance in green spaces and at the junction but fail to precisely detail the exact flood edge. Figure 2c displays a larger flood region than the actual image. In the city center, the forecasts almost entirely capture the flood region, and linear features like roads are clearly visible. Figure 2d covers a slightly larger area, including more of the flood region as shown in the actual images.

Despite underestimating the flood zone at the freeway junction, the second picture in the post-flood prediction demonstrates that the hue and extent in the city center nearly resemble the reference image. This may be the result of the post-event photograph from August 2016 which depicts a tiny quantity of floodwater still present in the city despite the fact that the flood level has greatly subsided, leaving only the remains of devastated trees and structures.

In the green, red, and NIR bands, Table 3 displays the RMSE and variance between actual and predicted Landsat readings. The projected findings’ noteworthy characteristics are shown in the table. First off, the August 29, 2016, Landsat and projected photos are all biased negatively. The predicted values produced by FSDAF seem to be inferior to those in the actual Landsat picture, despite the average discrepancies being tiny. As a result, the flood level or result determined by the prediction pictures may be exaggerated. Second, although the methods’ performance fluctuates in the red and green bands, it is consistent in the NIR band, with the RMSE of the NIR band in the FSDAF findings being constant at 0.04–0.07. (less than half of the RMSE in the green and red bands). Because most surface items (i.e., plants) reflect more light in the NIR band than in the visible bands, with the exception of water, which has a reflectance of almost zero, the increased prediction inaccuracy in the NIR band makes sense in floods. As a result, during a flood, the NIR band value may see a considerable decrease, which may occasionally result in a prediction mistake that is exaggerated in comparison to the other bands. In certain flooding mapping techniques, like NDWI, where the NIR band is employed as a crucial indicator to extract the flood zone, FSDAF findings may perform better in this respect. In agricultural areas, the Landsat pixel invariably blended agriculture with a small proportion of other vegetation categories or soil due to its medium spatial resolution. The blended pixel phenomenon was also observed in a few small parcels in agricultural areas. Additionally, the impact of shadows, terrain, and small clouds on flood inundation mapping was weak when data from the fused time sequence was added. Single Landsat spectral information may not provide enough data to accurately identify flood inundation boundaries and misclassifications in these regions, making it uncertain where the extent of flood damage lies. However, the fused time sequence information, which includes more flood information, is useful in identifying the extent of flood damage. By overcoming the spatial–temporal resolution trade-offs inherent in current satellite sensors, FSDAF contributes to the precision and effectiveness of flood monitoring systems. The ability to generate synthetic flood images with improved spatiotemporal resolutions allows for more accurate mapping of inundated areas during seasonal erosion flooding events.

**Table 3:** RMSE and Diff of the forecast value in the green, red, and NIR bands.

	2016.08.16	2016.08.30	2016.08.31	2016.09.01	2016.09.02	2016.09.03
Green RMSE	0.026	0.025	0.023	0.024	0.020	0.023
Green Diff*	− 0.016	− 0.013	− 0.013	− 0.009	− 0.004	− 0.007
Red RMSE	0.029	0.030	0.026	0.030	0.051	0.022
Red Diff*	− 0.014	− 0.014	− 0.011	− 0.013	− 0.001	− 0.005
NIR RMSE	0.128	0.171	0.110	0.048	0.090	− 0.082
NIR Diff*	− 0.060	− 0.002	− 0.031	− 0.016	− 0.003	− 0.010

*Diff: the mean difference between the predicted and observed values, *RMSE* root mean square error.

### Tumen river flood event simulation

The study involved simulating the Tumen River flood to determine the maximum submerged area, locations of drowned spots, and the time when the flood events occurred and stopped. To verify the accuracy of the simulation, a comparison was made with statistics obtained from the United Nations OCHA. Figure 2 shows several key findings: (1) the flood event occurred between August 29, 2016, and September 1, 2016; (2) the flood inundation had a similar cumulative pattern, with the maximum distribution on August 31, 2016, and the cumulative size increasing with the rainfall duration on the same underlying surface until September 1, 2016; (3) from September 2, 2016, the flood inundation surface distribution area began to decrease compared to the previous day.

The simulation model captured the submerged regions in places where the produced river network changed from the actual flow path, particularly where floods largely occurred due to river overflows. The simulated submersion regions in the upper part of the basin corresponded well with the investigated data, indicating that the imitation result was stable. Although the simulated submersion regions were similar to the practical flood as presented by the investigated data, there were still some inaccuracies in capturing the submersion regions in places where the produced river network deviated from the actual flow path. Despite FSDAF's remarkable performance in creating synthetic pictures for flood mapping, the geographical and temporal resolution of the input observation information may have a big influence on the outcomes. The resultant picture blending may be of low quality when there are few or no cloud-free remote-sensing photographs of the floods. Due to their independence from light and weather conditions, synthetic aperture radar (SAR) pictures have the potential to provide more accurate flooding assessment and monitoring^44^. Furthermore, the research demonstrates the potential of FSDAF in providing comprehensive information for studying land degradation and its correlation with flooding for sensitivity improvement in the measurement^45^. The insights gained from this research have broader implications for environmental monitoring for Transport of intensity diffraction^27,46^, sustainable land management, and disaster-related decision-making processes, emphasizing the importance of advanced remote sensing techniques in addressing complex challenges associated with dynamic environmental events.

The proposed Decision Tree categorization method, integrating Landsat OLI and MODIS spectral bands, brings both advantages and limitations to flood mapping. Its multi-sensor integration offers a comprehensive understanding of flood dynamics, but the model is sensitive to input quality and assumes homogeneity in flood characteristics. While it excels in temporal analysis, providing insights into post-flood impacts in urban settings, it may fall short in capturing dynamic changes and variations in water depth. Suggestions for improvement include incorporating high-resolution data, developing a dynamic modeling approach, validating with ground truth data, and utilizing additional sources for a more thorough post-flood analysis. These enhancements would bolster the model’s accuracy and broaden its applicability in advancing flood mapping technologies.

## Conclusion

The research on flexible spatiotemporal data fusion (FSDAF) for high-resolution flood mapping using Landsat and MODIS data holds significant implications for various fields, particularly in enhancing flood monitoring and disaster management. The forecasts may be enhanced by using the extra spectral data of flooding, resulting in better accuracy for post-flooding predictions. This is done by using post-flooding photos. The reflectance difference between flooded and non-flooded pixels is high enough to be distinguished by a DT classifier even if the FSDAF forecasts are inaccurate. In other words, when paired with post-event imagery, the inaccuracies in FSDAF predictions are too tiny to significantly misclassify flood inundation maps. As a consequence, when paired with post-flood photos, FSDAF projections may increase the accuracy of flood mapping. There are certain shortcomings in this study that will need to be addressed in the future. FSDAF may not be able to anticipate fast changes in urban floods adequately due to the spectral characteristics of flooding in urban areas and the complexity of the surface, which might lead to an under- or overestimation of the flooding area close to the flooding border. The mixing pattern may be altered to offer better forecasts of unexpected occurrences, leading to more precise flood mapping. More data, such as kinds of land cover, rainfall, elevation, sewage systems, the location of dams, and data on the city’s river gauge, may also be included to the classification process to increase the accuracy of flood mapping findings. This data may be used with hydrologic models to investigate surface damage and precisely map floods. Even though the studied data showed that the simulated inundation model was equivalent to the real flood, certain inundated areas were recorded in locations where the simulated river network diverged from the actual flow direction. A consequence map of the flooding submersion region is created during floods. The maximum submersion area and the seriously flooded regions are tested, and the start and end dates of the flood occurrence are also looked at.

## Data Availability

All data generated or analysed during this study are included in this published article.
